# Relationship between Urinary Liver-Type Fatty Acid-Binding Protein (L-FABP) and Sarcopenia in Spontaneously Diabetic Torii Fatty Rats

**DOI:** 10.1155/2020/7614035

**Published:** 2020-01-13

**Authors:** Jun Tanabe, Yuji Ogura, Keisei Kosaki, Yoshio Nagai, Takeshi Sugaya, Keiichi Ohata, Shiika Watanabe, Daisuke Ichikawa, Kazuho Inoue, Seiko Hoshino, Kenjiro Kimura, Seiji Maeda, Yugo Shibagaki, Atsuko Kamijo-Ikemori

**Affiliations:** ^1^Division of Nephrology and Hypertension, Department of Internal Medicine, St. Marianna University School of Medicine, Kanagawa, Japan; ^2^Department of Physiology, St. Marianna University School of Medicine, Kanagawa, Japan; ^3^Faculty of Health and Sport Sciences, University of Tsukuba, Ibaraki, Japan; ^4^Japan Society for the Promotion of Science, Tokyo, Japan; ^5^Division of Metabolism and Endocrinology, Department of Internal Medicine, St. Marianna University School of Medicine, Kanagawa, Japan; ^6^Department of Anatomy, St. Marianna University School of Medicine, Kanagawa, Japan; ^7^JCHO Tokyo Takanawa Hospital, Tokyo, Japan

## Abstract

**Background:**

Type 2 diabetes (T2D) is a known risk factor for diabetic kidney disease (DKD) and sarcopenia in older patients. Because there may be an interaction between DKD and sarcopenia, the aim of the present study is to investigate the relationship between urinary levels of liver-type fatty acid-binding protein (L-FABP) and sarcopenia using a novel rat model of T2D.

**Methods:**

Male spontaneously diabetic Torii (SDT) fatty rats (*n* = 5) at 16 weeks of age were used as an animal model of T2D. Age- and sex-matched Sprague-Dawley (SD) rats (*n* = 7) were used as controls. Urine samples were obtained from the rats, and muscle strength was evaluated with the use of the forelimb grip test at 16, 20, and 24 weeks of age. Serum, kidney, soleus, and extensor digitorum longus (EDL) muscle samples were collected at 24 weeks of age. Urinary L-FABP levels were measured using dedicated enzyme-linked immunosorbent assays.

**Results:**

Increased urinary L-FABP levels, focal glomerular sclerosis, moderate interstitial inflammation and fibrosis, and accumulation of renal oxidative proteins were significantly observed in the SDT fatty rats, compared to the SD rats. Muscle weight, muscle strength, cross-sectional areas of both type I and type IIb muscle fibers, and increasing rate of muscle strength were significantly decreased in the SDT fatty rats compared to the SD rats at 24 weeks. Urinary L-FABP levels at 20 and 24 weeks were significantly negatively correlated with muscle strength. Urinary L-FABP levels at 16 weeks were significantly negatively correlated with the increasing rate of muscle strength.

**Conclusions:**

Urinary L-FABP reflects the degree of muscle strength and weight, as well as cross-sectional areas of muscle fibers. Although further clinical study is needed, urinary L-FABP may be useful to monitor the progression of sarcopenia and DKD in T2D patients.

## 1. Introduction

Chronic kidney disease (CKD) is a common disease in aging societies worldwide. Many studies have focused on the involvement of CKD in the onset of sarcopenia which is characterized by decreased skeletal muscle mass and strength and has been the focus of many studies in order to sustain satisfactory quality of life and prevent fatal diseases in the super-aging populations of advanced countries [1–5].

Type 2 diabetes (T2D) with insulin resistance is a common risk factor for diabetic kidney disease (DKD) and sarcopenia [1, 2]. Furthermore, there may be an association between the development of DKD and sarcopenia. Therefore, comprehensive medical management of T2D to prevent both DKD and sarcopenia is needed in clinical practice. However, at present, there are no universally accepted markers to monitor the degree of sarcopenia in DKD patients.

There is a possibility that exercise not only is indispensable for the prevention of sarcopenia but may also be useful to inhibit the progression of DKD. Although the effects of aerobic training on renal morphometric abnormalities in a Zucker fatty rat model of impaired glucose tolerance remains controversial [3], a recent study with the Zucker diabetic fatty rat reported that chronic exercise more certainly prevented interstitial profibrotic change rather than glomerular sclerosis via decreased oxidative stress and improved renal microcirculation by upregulation of endothelial nitric oxide synthase expression [4]. Another study demonstrated the renal antifibrosis effects of exercise by promoting skeletal muscle growth in a model of advanced renal interstitial fibrosis [5]. A recent study by our group revealed that tubulointerstitial damage due to inappropriate activation of the renin-angiotensin-aldosterone system was attenuated by voluntary running exercise [6]. Based on these results, we hypothesize that there may be some sort of relationship between tubulointerstitial damage and sarcopenia.

Urinary liver-type fatty acid-binding protein (L-FABP) accurately reflects the degree of tubulointerstitial damage [7, 8] and has been accepted as a tubular marker by the Ministry of Health, Labour and Welfare in Japan [9]. Moreover, a study by our group found that urinary L-FABP levels were inversely associated with exercise capacity or physical activity and that aerobic exercise training decreased urinary L-FABP levels in healthy middle-aged and older adults [10, 11]. Therefore, urinary L-FABP may be useful to monitor the progression of sarcopenia.

Among the limited models of both DKD and sarcopenia, the spontaneously diabetic Torii (SDT) fatty rat is a unique experimental novel model of T2D with kidney disease and sarcopenia [12–14] that mimics the pathophysiology of human T2D. Therefore, the aim of the present study was to elucidate the relationship between urinary L-FABP and sarcopenia using the SDT fatty rat.

## 2. Materials and Methods

### 2.1. Animals

All animal studies were conducted in strict accordance with the St. Marianna University School of Medicine Institutional Guide for Animal Experiments and the Guide for the Care and Use of Laboratory Animals. All surgery was performed under 3% isoflurane anesthesia, and all efforts were made to minimize suffering.

In this study, male SDT fatty (SDT.Cg-*Lepr*^fa^/JttJcl) rats, derived from a Sprague-Dawley (SD) colony, were used as a model of T2D. Five-week-old male SDT fatty rats (*n* = 5) and SD rats (*n* = 7) of the same age (controls) were purchased from CLEA Japan, Inc. (Tokyo, Japan) and housed under standard conditions with free access to laboratory chow (CRF-2, Charles River Laboratories Japan, Inc., Yokohama, Japan) and water. Body weight, chow consumption, and muscle strength were measured every 4 weeks from 16 to 24 weeks of age, which is equivalent to a late middle-aged human patient. Urine was collected from the rats using metabolic cages. Muscle strength was measured using the forelimb grip test with a grip strength meter (MK-380CM/FM; Muromachi Kikai, Co., Ltd., Tokyo, Japan). The average of three measurements of muscle strength per animal per time point was recorded for comparative analysis.

For the experiments, 16-, 20-, and 24-week-old rats were individually housed overnight in metabolic cages with free access to tap water. Urine was collected for the measurement of urinary markers. Additionally, serum samples, leg muscle tissues, and kidney tissues were collected from rats that were sacrificed at the age of 24 weeks. The extracted kidneys were weighted and cut into pieces for various analyses. The extracted muscles specimens were sectioned into the soleus muscle as a slow muscle and the extensor digitorum longus (EDL) muscle as a fast muscle. After weighing, each muscle was frozen individually in liquid nitrogen and stored at -80°C for further analyses.

### 2.2. Blood Pressure Measurement

Systolic blood pressure (SBP) was measured using a tail-cuff apparatus (Softron BP-98A; Softron, Co., Ltd., Tokyo, Japan) every 4 weeks from 16 to 24 weeks of age. The average of three measurements of SBP per animal per time point was recorded for analysis.

### 2.3. Serum and Urinary Biochemistry

Serum and urine creatinine levels were measured using a QuantiChrom™ Creatinine Assay Kit (BioAssay Systems LLC, Hayward, CA, USA). Serum cystatin C levels were measured using a rat cystatin C enzyme-linked immunosorbent assay (ELISA) kit (BioVendor Instruments a.s., Brno, Czech Republic). Serum insulin (FUJIFILM Wako Shibayagi Corporation, Shibukawa, Japan) and insulin-like growth factor-1 (IGF-1; R&D Systems, Inc., Minneapolis, MN, USA) levels were measured using dedicated ELISAs. Serum urea nitrogen levels were measured via urease ultraviolet absorption spectrophotometry, serum total cholesterol levels were measured using the cholesterol oxidase enzymatic method, and serum triglyceride levels were measured using an enzymatic method conducted by SRL Clinical Laboratory Testing Services (Tokyo, Japan). Urinary glucose levels were determined semiquantitatively using urinary dipsticks (Wako Pure Chemical Industries, Ltd., Osaka, Japan). Urinary glucose was graded as follows: 0, negative; 1+, >100 mg/dl; 2+, >250 mg/dl; 3+, >500 mg/dl; and 4+, >2000 mg/dl.

Urinary levels of L-FABP and albumin were measured using rat L-FABP (CMIC Co., Ltd., Tokyo, Japan) and rat albumin (Exocell, Inc., Philadelphia, PA, USA) ELISA kits, respectively. Urinary levels of rat L-FABP and albumin were reported as ratios relative to urinary creatinine levels.

### 2.4. Renal Histological and Morphometric Analysis

The midsection of each excised kidney was dissected along the minor axis. The two pieces were fixed in 10% buffered formalin or methyl Carnoy's solution (60% methanol, 30% chloroform, and 10% glacial acetic acid) and embedded in paraffin. Serial sections (3 *μ*m thickness) were prepared for renal immunohistochemical assessment and periodic acid-Schiff (PAS) staining.

Glomerulosclerosis in PAS-stained sections was evaluated by grading the extent of sclerosis in each glomerulus as follows: 0, no sclerosis; 1, sclerosis in 1%–25% of the glomerulus; 2, sclerosis in 26%–50% of the glomerulus; 3, sclerosis in 51%–75% of the glomerulus; and 4, sclerosis in 76%–100% of the glomerulus. At 100x magnification, the glomerulosclerosis score for each animal was calculated as follows: (1 × the number of grade 1 glomeruli, %) + (2 × the number of grade 2 glomeruli, %) + (3 × the number of grade 3 glomeruli, %) + (4 × the number of grade 4 glomeruli, %). Fifty glomeruli were examined for each animal [15].

### 2.5. Renal Immunohistochemical Analysis

Target antigens in the preprocessed sections were stained using the indirect immunoperoxidase method, as described previously [7]. Briefly, formalin-fixed, paraffin-embedded tissue specimens were used to stain for myofibroblasts and tubular cells exhibiting epithelial-mesenchymal transition using a mouse monoclonal antibody specific for *α*-smooth muscle actin (*α*-SMA; dilution, 1 : 800; Sigma-Aldrich Corporation, St. Louis, MO, USA). The tissue specimens fixed in methyl Carnoy's solution were assessed immunohistochemically for macrophages using a mouse monoclonal antibody (ED-1) specific for CD68 (1 : 100; Abcam, Tokyo, Japan) and goat polyclonal antibodies specific for type I and III collagen (1 : 200; Southern Biotech, Birmingham, AL, USA). Labeled proteins were visualized using polymeric horseradish peroxidase-conjugated secondary antibodies (ImmPRESS™ Polymer Detection Kit; Vector Laboratories, Burlingame, CA, USA). Peroxidase activity was detected via the diaminobenzidine reaction (Liquid DAB+; DAKO Japan, Inc., Tokyo, Japan), and sections were counterstained with hematoxylin. For quantification, images from 10 nonoverlapping fields throughout the cortical and outer medullary regions were captured at 100x magnification. The extent of macrophage and myofibroblast infiltration of the cortical and outer medullary interstitia was automatically measured using an image analyzer (WinRoof version 6.4; Mitani Corporation, Tokyo Japan). Briefly, the areas positively stained for CD68 and *α*-SMA were independently measured and expressed as ratios relative to the areas of the entire cortical and outer medullary regions. The same method was used to measure the expression levels of type I and III collagen.

### 2.6. Immunohistochemical Analysis of Muscle Tissues

The frozen excised soleus and EDL muscle tissues were cut centrally along the width and embedded in Tissue-Tek® O.C.T.™ Compound (Sakura Finetek Japan Co., Ltd., Tokyo, Japan). Continuous frozen sections sliced to a thickness of 10 *μ*m were prepared using a cryostat (HM 550; Thermo Fisher Scientific, Rockford, IL, USA). Double-color immunofluorescent staining was performed in order to evaluate the diameter of each type I and IIb fiber.

The dried sections were washed three times with phosphate-buffered saline (PBS) for 5 min each time and soaked in methanol solution containing 0.3% hydrogen peroxide for 20 min. After washing three times with PBS for 5 min again, the sections were incubated with the following primary antibodies at 4°C overnight: anti-type I myosin heavy chain (MyHC) isoform IgG2b (MyHCI; 1 : 200; BA-F8; Developmental Studies Hybridoma Bank (DSHB), the University of Iowa, Iowa City, IA, USA), anti-type IIb MyHC isoform immunoglobulin (Ig)G1 (MyHCIIb; 1 : 100; F18; DSHB), and anti-laminin IgG2a (1 : 200; 2E8; DSHB). After washing three times with PBS, the sections were incubated with the following fluorescent-labeled secondary antibodies at room temperature for 1 h: Alexa Flour 488-labeled goat anti-mouse IgG2b (1 : 500; Invitrogen Corporation, Carlsbad, CA, USA), DyLight 405-labeled goat anti-mouse IgG1 (1 : 500; Invitrogen Corporation), and Alexa Flour 488-labeled goat anti-mouse IgG2a (1 : 500; Invitrogen Corporation).

For quantification, tissue images of the soleus and EDL muscles were obtained using ZEN 2 pro imaging software (Carl Zeiss Microscopy Co., Ltd., Tokyo, Japan). The cross-sectional areas of the specimens were evaluated using 300 fibers in two visual fields at 100x magnification per animal using an image analyzer (WinRoof).

### 2.7. Western Blot Analysis

Proteins were extracted from the collected muscle tissues and concentrations were measured as described previously [16]. Next, 30 *μ*g of extracts were separated by sodium dodecyl sulfate-polyacrylamide gel electrophoresis using NuPAGE 4%–12% Bis-Tris gels and the XCell SureLock Mini-Cell system (Thermo Fisher Scientific). The separated proteins were transferred to polyvinylidene difluoride membranes using the iBlot™ dry blotting system (Thermo Fisher Scientific). The membranes were blocked with Blocking One solution (Nacalai Tesque, Inc., Kyoto, Japan) and then incubated overnight at 4°C with primary antibodies diluted in Can Get Signal Solution I (TOYOBO, Osaka, Japan) against Akt (rabbit monoclonal; #9272; 1 : 1000; Cell Signaling Technology, Inc., Danvers, MA, USA), phospho-Akt (Ser473) (rabbit monoclonal; #9271; 1 : 1000; Cell Signaling Technology, Inc.), mammalian target of rapamycin (mTOR; rabbit monoclonal; #2983; 1 : 1000; Cell Signaling Technology, Inc.), phospho-mTOR (Ser2448) (rabbit monoclonal; #5536; 1 : 1000; Cell Signaling Technology, Inc.), ribosomal protein S6 kinase beta-1 (S6K; rabbit monoclonal; #2708; 1 : 1000; Cell Signaling Technology, Inc.), phospho-S6K (rabbit monoclonal; #9234; 1 : 1000; Cell Signaling Technology, Inc.), 5′AMP-activated protein kinase (AMPK; rabbit monoclonal; #2603; 1 : 1000; Cell Signaling Technology, Inc.), phospho-AMPK (Thr172; rabbit monoclonal; #2535; 1 : 1000; Cell Signaling Technology, Inc.), Forkhead box protein O1 (FoxO1; rabbit monoclonal; #9454; 1 : 1000; Cell Signaling Technology, Inc.), phospho-FoxO1 (Ser256) (rabbit monoclonal; #9461; 1 : 1000; Cell Signaling Technology, Inc.), Atrogin-1 (rabbit polyclonal; 1 : 1000; ECM Biosciences, Versailles, KY, USA), and muscle RING-finger protein-1 (MuRF-1; rabbit polyclonal; 1 : 1000; ECM Biosciences). After washing, the membranes were incubated with a horseradish peroxidase-conjugated anti-rabbit antibody (Santa Cruz Biotechnology, Inc., Dallas, TX, USA) diluted at 1 : 2000 in Blocking One solution for 1 h at room temperature. Subsequently, chemiluminescence was detected using an ECL Prime western blotting detection reagent (GE Healthcare, Little Chalfont, UK), and images were obtained with a charge-coupled device camera system (ImageQuant LAS4000; GE Healthcare Japan, Tokyo, Japan).

The ratios of phosphorylated to total proteins were quantitated by densitometry analysis of immunoblots using ImageJ software (https://imagej.nih.gov/ij/). The same membranes that were incubated with antibodies against FoxO1 and phospho-FoxO1A were also incubated with rabbit monoclonal anti-*α*-tubulin antibody (1 : 4000; Abcam). After treatment with a stripping buffer (Wako), FoxO1 protein expression was normalized to that of *α*-tubulin.

### 2.8. Evaluation of Oxidative Stress in Muscle and Kidney Tissues

After determining protein concentrations, to evaluate oxidative stress in the muscle and kidney tissues, protein carbonyls, which are the most common products of protein oxidation, were quantified using an OxiSelect™ Protein Carbonyl ELISA Kit (Cell Biolabs, Inc., San Diego, CA, USA) with 100 *μ*g of protein extracted from both tissue types.

### 2.9. Statistical Analysis

All values are expressed as the mean ± standard error of the mean (SEM). A probability (*p*) value of <0.05 was considered statistically significant. Following the Kruskal-Wallis test, differences among each SD and SDT rat were identified using the Steel-Dwass test. The Mann-Whitney *U* test was used for comparisons between two groups. Spearman's rank correlation coefficient was used to evaluate nonparametric data and assess correlations between two parameters. Changes in muscle strength of each rat throughout the experimental period were defined as the slope of the regression line of a line-graph plot of muscle strength against experimental duration (weeks). All statistical analyses were performed using JMP® software, version 13.0.0 (SAS Institute, Cary, NC, USA).

## 3. Results

### 3.1. Changes in Body Weight, Blood Glucose Level, SBP, and Food Intake

The body weight, blood glucose level, SBP, and food intake of all rats in both groups were measured every 4 weeks between the ages of 16 and 24 weeks. As shown in Table 1, while the mean body weight in the SD group was significantly greater at 24 weeks than 16 weeks, that of the SDT group increased, but not significantly. There were no significant differences in the body weights between the two groups throughout the experimental period. Blood glucose levels were significantly higher in the SDT group than the SD group throughout the experimental period. Although there was no change over time in the SD group, SBP levels in the SDT group moderately increased throughout the experimental period. Food intake increased significantly at 20 and 24 weeks compared to 16 weeks in the SD group. Additionally, food intake at 16 and 20 weeks was significantly greater in the SDT group than the SD group.

### 3.2. Comparison of Kidney Function and Serum Parameters at 24 Weeks of Age

As shown in Table 2, at 24 weeks, serum creatinine and urea nitrogen levels were significantly increased in the SDT group compared to the SD group, while there was no significant difference in serum cystatin C levels. Serum insulin levels were comparable between the two groups, while serum IGF-1 levels were significantly lower in the SDT group. Both serum total cholesterol and triglyceride levels were significantly greater in the SDT group than the SD group.

### 3.3. Changes in Urinary Glucose, L-FABP, and Albumin Levels

**Figure 1:**
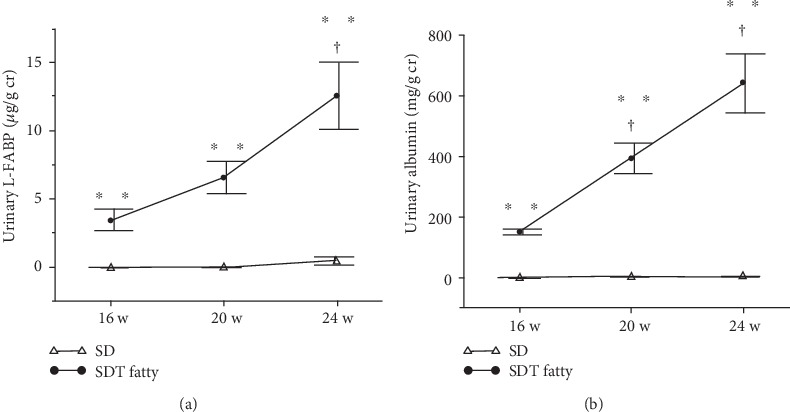
Changes in urinary parameters of SD and SDT fatty rats. Urinary L-FABP levels at 16, 20, and 24 weeks of age (a) and urinary albumin levels at 16, 20, and 24 weeks of age (b). Values are presented as the mean ± standard error of the mean (SEM). ^∗∗^*p* < 0.01 vs. SD rats at the same age; ^†^*p* < 0.05 vs. SDT fatty rats at 16 weeks of age.

The urinary glucose scores of the SDT and SD groups at 16, 20, and 24 weeks remained unchanged at 4 and 0, respectively. In the SD group, urinary L-FABP (Figure 1(a)) and urinary albumin (Figure 1(b)) levels remained unchanged throughout the experimental period. Conversely, urinary L-FABP at 24 weeks (Figure 1(a)) and urinary albumin at both 20 and 24 weeks (Figure 1(b)) were significantly increased in the SDT group compared to 16 weeks. Also, urinary L-FABP (Figure 1(a)) and urinary albumin (Figure 1(b)) levels were significantly higher in the SDT group than the SD group throughout the experimental period.

### 3.4. Immunohistological Analysis of Kidney Tissues

**Figure 2:**
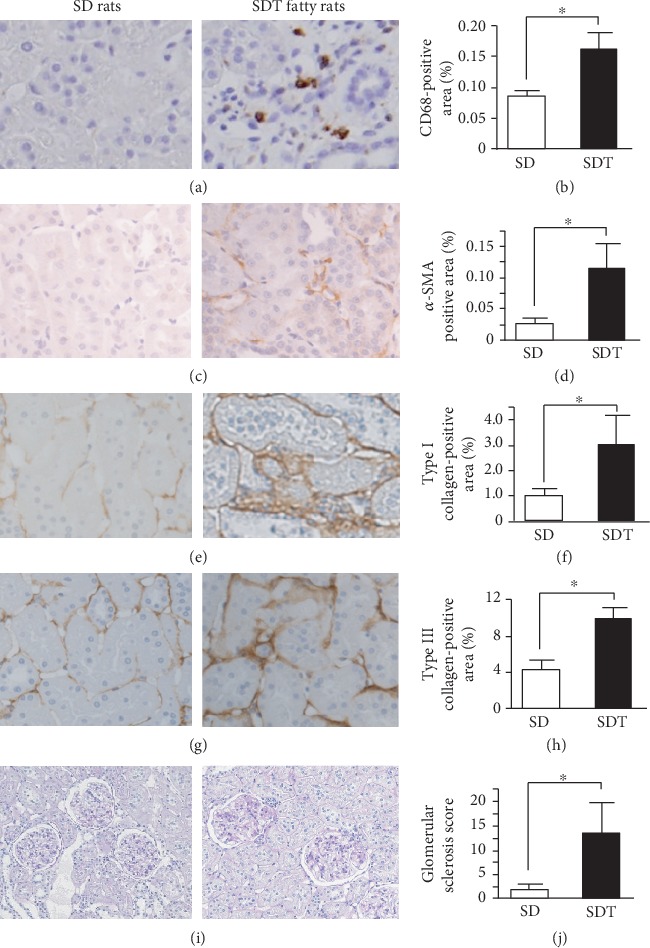
Histological evaluation of the kidney in SD and SDT fatty rats. Immunohistological staining of kidney tissues using antibodies against CD68 (a, b), *α*-SMA (c, d), type I collagen (e, f), and type III collagen (g, h). Histological PAS staining showing focal glomerular sclerosis (i) and semiquantitative assessment of focal glomerular sclerosis (j). Original magnification: ×100. Values are presented as the mean ± SEM. ^∗^*p* < 0.05 vs. SD rats.

To evaluate the degree of renal interstitial inflammation, immunohistochemical analysis of the macrophage marker CD68 was conducted. Macrophages were detected in the interstitium of the SDT fatty rats (Figure 2(a)), and the degree of macrophage infiltration was significantly greater in the SDT group than the SD group (Figure 2(b)).

Additionally, immunohistochemical analyses of *α*-SMA and type I and III collagen were performed to evaluate the extent of tubulointerstitial fibrosis. Areas positive for *α*-SMA were observed in the tubules and interstitium of the SDT group (Figure 2(c)) to a significantly greater extent compared to the SD group (Figure 2(d)). Further analysis revealed that the expression levels of type I (Figures 2(e) and 2(f)) and III (Figures 2(g) and 2(h)) collagen were significantly greater in the SDT group than the SD group.

### 3.5. Evaluation of Glomerular Sclerosis

The mean glomerular sclerosis score was significantly greater for the PAS-stained kidney tissues of the SDT group than the SD group throughout the experimental period (Figures 2(i) and 2(j)).

### 3.6. Changes in Muscle Strength

**Figure 3:**
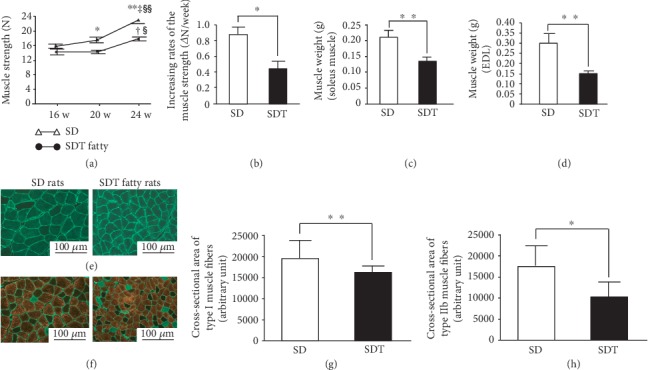
Evaluation of muscle strength, muscle weight, and muscle histological analysis. Change in muscle strength in SD and SDT fatty rats (a) and increasing rates of muscle strength (b). Weight of lower limb muscles in SD and SDT fatty rats. Soleus muscle (c) and EDL muscle (d) at 24 weeks of age. Multiple fluorescent staining of soleus muscle fibers (e) and semiquantitative assessment of type I fibers in the soleus muscle (g). Multiple fluorescent staining of EDL muscle fibers (f) and semiquantitative assessment of type IIb fibers in EDL muscle (h). Type I fibers are stained green using antibodies against MyHCI and type IIb fibers are stained red using antibodies against MyHCIIb. The frame of the fibers stained green show laminin. Original magnification: ×100. Values are presented as the mean ± SEM. ^∗^*p* < 0.05 and ^∗∗^*p* < 0.01 vs. SD rats at the same age; ^†^*p* < 0.05 and ^‡^*p* < 0.01 vs. the same group at 16 weeks of age; ^§^*p* < 0.05 and ^§§^*p* < 0.01 vs. the same group at 20 weeks of age.

Muscle strength significantly increased in the SD and SDT groups at 24 weeks compared to 16 and 20 weeks but was significantly lower in the SDT group compared to the SD group at 20 and 24 weeks (Figure 3(a)). The rate of increased muscle strength was significantly lower in the SDT group than the SD group (Figure 3(b)).

### 3.7. Comparison of Muscle Weight and Immunohistological Analysis of Muscle Tissues

The muscle weights of the soleus and EDL muscles were significantly lower in the SDT group than the SD group (Figures 3(c) and 3(d)). Since the soleus is mainly composed of slow-twitch muscle fibers [17], a cross-sectional area of type I muscle fibers was evaluated (Figure 3(e)). The EDL muscle has a high proportion of fast-twitch muscle fibers [17]; thus, a cross-sectional area of type IIb muscle fibers was evaluated (Figure 3(f)). Type I muscle fiber was stained green (Figure 3(e)) and type IIb muscle fiber was stained red (Figure 3(f)). The cross-sectional areas of muscle fiber types I (Figure 3(g)) and IIb (Figure 3(h)) were significantly decreased in the SDT group compared to the SD group.

### 3.8. Evaluation of Muscle Protein Synthesis or Degradation

Maintenance of muscle mass is regulated by the balance between protein synthesis and degradation. Hence, the activation of insulin/IGF-1-Akt and mTOR-S6K signaling as markers of protein synthesis and the activation of insulin/IGF-1-Akt-FoxO signaling, phosphorylation of AMPK, and expression of E3 ubiquitin ligases as markers of protein degradation were evaluated.

**Figure 4:**
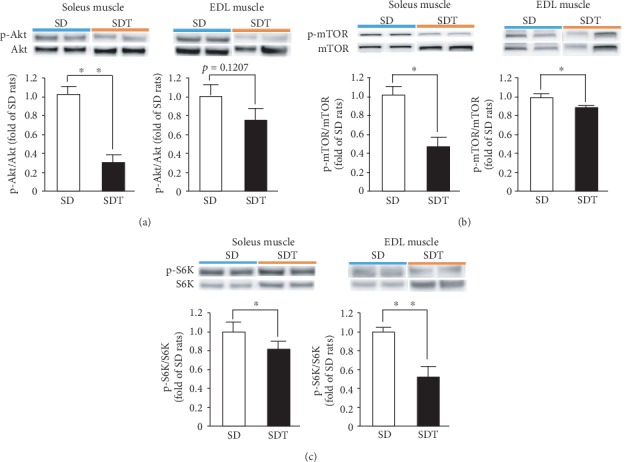
Muscle protein synthesis in SD and SDT fatty rats, as determined by western blot analysis. Phosphorylated Akt (a), mTOR (b), and S6K (c) in the soleus and EDL muscles. ^∗^*p* < 0.05 and ^∗∗^*p* < 0.01 vs. SD rats.

**Figure 5:**
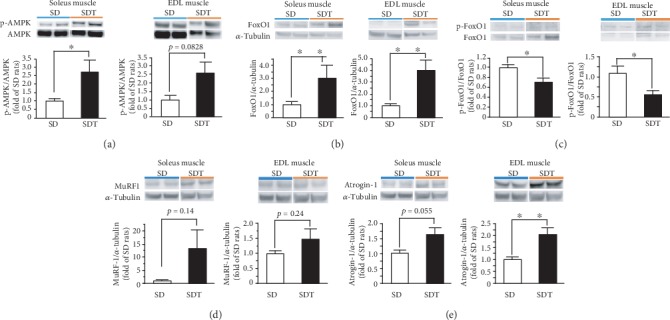
Muscle protein degradation in SD and SDT fatty rats, as determined by western blot analysis. Phosphorylated AMPK in the soleus and EDL muscles (a). Protein expression of FoxO1 in the soleus and EDL muscles (b). Phosphorylated FoxO1 in the soleus and EDL muscles (c). Protein expression of the E3 ubiquitin ligases MuRF-1 (d) and Atrogin-1 (e) in SD and SDT fatty rats, as determined by western blot analysis. ^∗^*p* < 0.05 and ^∗∗^*p* < 0.01 vs. SD rats.

The phosphorylation levels of Akt (Figure 4(a)), mTOR (Figure 4(b)), and S6K (Figure 4(c)) were significantly lower, while that of AMPK (Figure 5(a)) was significantly higher in the soleus muscle of the SDT group compared to the SD group. Regarding the EDL muscle, although the phosphorylation levels of both Akt (Figure 4(a)) and AMPK (Figure 5(a)) were not significantly different between the SDT and SD groups, those of mTOR (Figure 4(b)) and S6K (Figure 4(c)) were significantly lower in the SDT group.

FoxO1 protein expression was significantly higher (Figure 5(b)), while that of phospho-FoxO1 was significantly lower, in both the soleus and EDL muscles of the SDT group compared to the SD group (Figure 5(c)).

Regarding the protein expression profiles of E3 ubiquitin ligases (Figures 5(d) and 5(e)), Atrogin-1 (Figure 5(e)) expression levels were significantly higher in the EDL muscle of the SDT group than the SD group, but not MuRF-1 (Figure 5(d)). There was no significant change in Atrogin-1 and MuRF-1 expression levels in the soleus muscle between the SDT and SD groups (Figures 5(d) and 5(e)).

### 3.9. Correlations between Each Urinary Marker and Muscle Strength

**Figure 6:**
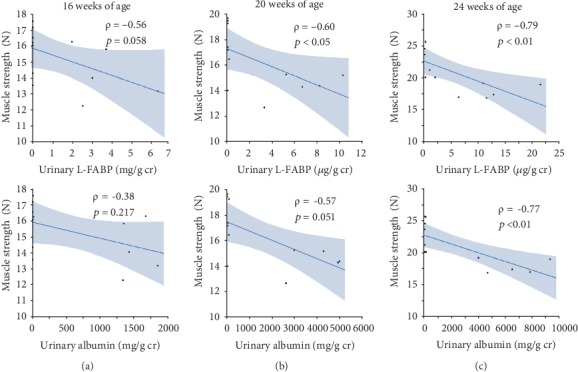
Correlations between each urinary marker and muscle strength at 16 (a), 20 (b), and 24 weeks of age (c).

Urinary L-FABP levels were inversely correlated with muscle strength (*ρ* = −0.34, *p* < 0.05), but not urinary albumin, throughout the experimental period (*ρ* = −0.23, *p* = 0.188). At 16 weeks, urinary L-FABP and albumin levels were not significantly correlated with muscle strength (Figure 6(a)). At 20 and 24 weeks, while urinary L-FABP levels were inversely correlated with muscle strength (Figures 6(b) and 6(c)), urinary albumin levels were inversely correlated with muscle strength only at 24 weeks (Figure 6(c)).

**Figure 7:**
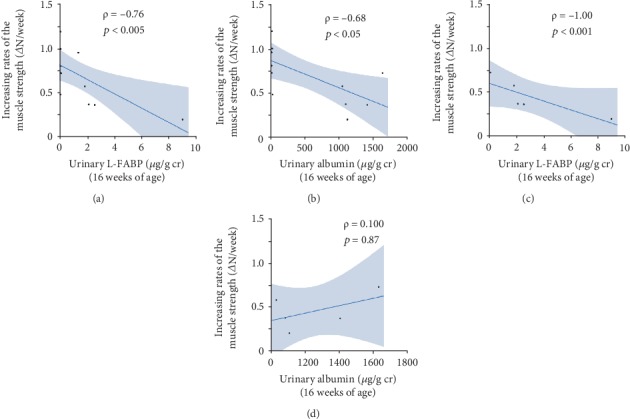
Correlations between each urinary marker at 16 weeks of age and increasing rate of muscle strength from 16 to 24 weeks of age in SD and SDT fatty rats (a, b). Correlations between each urinary marker and increasing rate of muscle strength from 16 to 24 weeks of age in SDT fatty rats (c, d).

Urinary L-FABP (*ρ* = −0.76, *p* < 0.005, Figure 7(a)) and albumin (*ρ* = −0.68, *p* < 0.05, Figure 7(b)) levels at 16 weeks were inversely correlated with an increasing rate of muscle strength. In the SDT group at 16 weeks, urinary L-FABP levels were inversely correlated with an increasing rate of muscle strength (*ρ* = −1.00, *p* < 0.001, Figure 7(c)), but not urinary albumin levels (*ρ* = 0.10, *p* = 0.87, Figure 7(d)). Regarding urinary L-FABP, the significance was maintained even after removing an extreme deviation from the mean, as shown in Figures 7(a) and 7(c).

### 3.10. Correlation between Urinary L-FABP and Muscle Weight, and Cross-Sectional Areas of Both Type I and IIb Muscle Fibers

**Figure 8:**
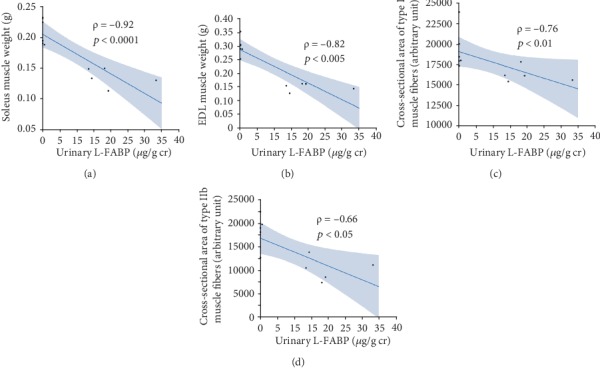
Correlations of urinary L-FABP with the weights of the soleus (a) and ELD muscles (b) and cross-sectional areas of type I (c) and IIb (d) muscle fibers.

Urinary L-FABP levels at 24 weeks were inversely correlated with the weight of the soleus (*ρ* = −0.92, *p* < 0.0001, Figure 8(a)) and EDL (*ρ* = −0.82, *p* < 0.005, Figure 8(b)) muscles, as well as cross-sectional areas of both type I (*ρ* = −0.76, *p* < 0.01, Figure 8(c)) and IIb (*ρ* = −0.66, *p* < 0.05, Figure 8(d)) muscle fibers.

### 3.11. Correlation between Urinary L-FABP and Urinary Albumin, and Glomerular Sclerosis Score

**Figure 9:**
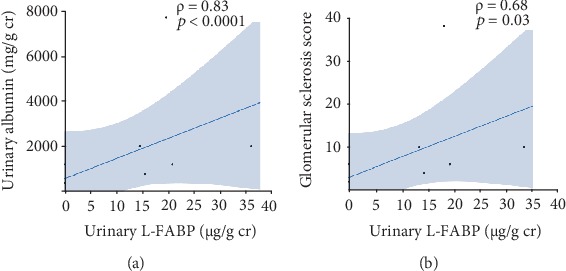
Correlations of urinary L-FABP with urinary albumin (a) and glomerular sclerosis (b).

Urinary L-FABP levels were significantly correlated with both urinary albumin (*ρ* = 0.83, *p* < 0.0001, Figure 9(a)) and glomerular sclerosis scores (*ρ* = 0.68, *p* < 0.05, Figure 9(b)).

### 3.12. Correlation between Renal Morphological Change and Muscle Strength and Weight

The degrees of macrophage infiltration and *α*-SMA expression at 24 weeks were inversely correlated with the strength and weight of the soleus and EDL muscles (Table 3). Type I and III collagen expressions were inversely correlated with the weights of the soleus and EDL muscles, but not strength (Table 3). Glomerular sclerosis score was inversely correlated with the strength and weight of EDL muscles, but not the weight of the soleus (Table 3).

### 3.13. Evaluation of Oxidative Stress in Kidney and Muscle Tissues

**Figure 10:**
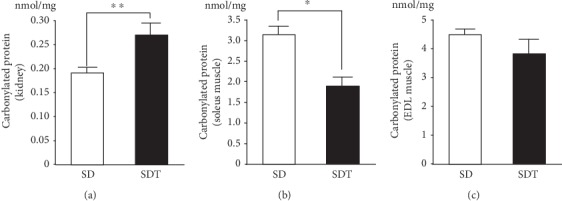
Expression profiles of carbonylated proteins in kidney (a), soleus (b), and EDL muscle (c) tissues. Values are presented as the mean ± SEM. ^∗^*p* < 0.05 and ^∗∗^*p* < 0.01 vs. SD rats.

While protein carbonyl levels in kidney tissues were significantly higher in the SDT group (Figure 10(a)), those in the soleus muscle, but not the EDL muscle, were significantly lower compared to those in the SD group (Figures 10(b) and 10(c)).

## 4. Discussion

The results of the present study revealed that urinary L-FABP secretion reflected decreases in muscle strength, muscle weight, and cross-sectional areas of muscle fibers, and the high levels of urinary L-FABP at 16 weeks were associated with a lower increasing rate of muscle strength in mature SDT fatty rats with hyperglycemia, hyperlipidemia, low levels of serum IGF-1, and kidney disease. The current study indicated that urinary L-FABP might be useful to monitor the progression of sarcopenia in addition to DKD in T2D patients.

There is growing evidence of a correlation between sarcopenia and kidney disease, including DKD [18]. The results of the present study indicated a correlation between urinary markers and sarcopenia in mature rats with T2D and DKD. Our previous studies found that lower physical activity or weaker muscle strength was related to increased urinary L-FABP levels, but not urinary albumin [10], and that intervention with aerobic exercise training facilitated a reduction in increased urinary L-FABP in healthy middle-aged and older adults [11]. Furthermore, decreased muscle strength was independently associated with increased intrarenal vascular resistance [19], suggesting that sarcopenia might lead to impairments to the intrarenal vasculature. Considering that increased urinary L-FABP is induced by decreased renal microvascular blood flow [20] and that renal hypoxia was observed in the SDT fatty rats with DKD (unpublished data), urinary L-FABP might be useful for the detection of abnormalities in renal hemodynamics aggravated by sarcopenia in addition to glomerular sclerosis leading to renal hypoxia derived from reduction of postglomerular blood flow. On the contrary, accumulation of uremic toxins, metabolic acidosis, and anemia due to deterioration of renal function is reported to lead to sarcopenia [18]. Although renal dysfunction was mild in the SDT fatty rats in the present study, muscle mass loss was reported to appear in the early phase of CKD [21]. Therefore, in the present study, DKD might have influenced the progression of sarcopenia.

The progression of sarcopenia in T2D patients has been associated with lower renal function and higher urinary albumin levels [22]. As a marker of the severity of kidney disease, urinary albumin may reflect the degree of muscle weakness in patients with kidney disease. In the present study, urinary albumin at 16 weeks was significantly related to continued muscle growth in all rats used in the study, although the significant correlation disappeared in only SDT fatty rats. Hence, further studies are needed to determine whether urinary albumin, like urinary L-FABP, is a risk factor for the onset of sarcopenia.

In the skeletal muscle, there are four major kinds of muscle fiber: type I, type IIa, type IIx, and type IIb. The predominance of one over the other is dependent on the contractile rate and metabolic characteristics. While type I fibers, which are slow-twitch and oxidative fibers, are influenced by insulin resistance in T2D [23], type IIb fibers, which are fast-twitch and glycolytic fibers, are influenced by aging [24]. Although serum insulin levels were similar in the SDT and SD groups, blood glucose levels were significantly higher in the SDT group, suggesting the presence of insulin resistance. Regarding the influence of muscle fiber on kidney disease, CKD induced insulin resistance [25], leading to a decrease in the proportion of type I fibers, as previously shown in the gastrocnemius muscle of a CKD model [26, 27]. In the present study, type I fibers in the soleus muscle and type IIb fibers in the EDL muscle were assessed separately, which revealed decreases in both muscle weight and cross-sectional areas of type I and IIb fibers in the present study. Based on these results, insulin resistance, aging, and DKD might be related to the progression of sarcopenia in SDT fatty rats.

**Figure 11:**
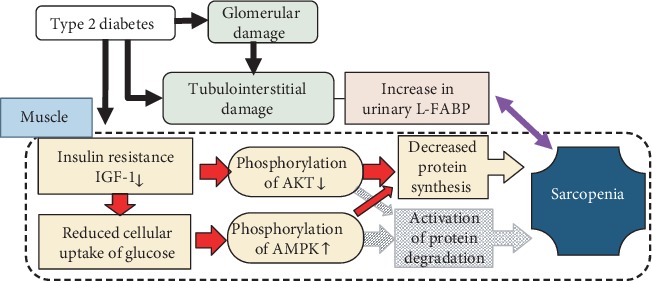
Molecular mechanism underlying the progression of sarcopenia in type 2 diabetes. Disruption to insulin/IGF-1-Akt signaling due to insulin resistance and low serum IGF-1 levels was considered to reduce muscle protein synthesis and to provoke abnormalities in muscle strength and mass in the SDT fatty rats. Furthermore, phosphorylation of AMPK due to reduced cellular uptake of glucose led to suppression of muscle protein synthesis. On the other hand, the involvement of muscle protein degradation signaling in muscle depression in the SDT group may be low.

Insulin or the IGF-1 signaling pathway plays a critical role in the regulation of muscle mass by controlling the balance between protein synthesis and degradation in muscle [28]. As a critical downstream effector of the insulin/IGF-1 pathway, phosphorylation of Akt leads to activation of protein synthesis via phosphorylation of both mTOR and S6K or inhibition of protein degradation via FoxO1 phosphorylation, which results in downregulated expression levels of the E3 ligases Atrogin-1 and MuRF-1 [28]. Furthermore, AMPK, which is known to be a cellular fuel sensor activated by ATP depletion, is related to suppression of protein synthesis via decreased phosphorylation of mTOR [29] and activation of the FoxO-dependent protein degradation pathway [30]. In the present study, phosphorylation of Akt decreased and that of AMPK increased in the soleus muscle, but not in the EDL muscle, in the SDT fatty rats. Phosphorylation of mTOR, S6K, and FoxO1 was reduced in both the soleus and EDL muscles. Although increased Atrogin-1 expression was observed in the EDL muscle, there was no significant difference in MuRF-1 expression in the soleus and EDL muscles between the SDT and SD groups. Regarding the molecular mechanism underlying the progression of sarcopenia in the SDT group, disruption to insulin/IGF-1-Akt signaling due to insulin resistance and low serum IGF-1 levels was considered to provoke abnormalities in muscle strength and mass in the SDT group (Figure 11). Furthermore, while decreased muscle protein synthesis was considered to contribute to weakness of both the soleus and EDL muscles in the SDT group, the involvement of muscle protein degradation signaling in muscle depression in the SDT group may be low (Figure 11). Although the results of this study support those of a previous study [14], further studies are needed to investigate the association of FoxO-associated downstream pathways with muscle depression in SDT fatty rats.

Oxidative stress is reported to promote muscle atrophy and weakness [31–33] in addition to CKD. However, decreased oxidative stress, as evaluated by carbonylation of muscle proteins, was observed in the soleus muscle of SDT fatty rats, despite increased oxidative stress in the kidney tissues. Antioxidants are reported to diminish the positive effects of exercise training via decreased generation of reactive oxidative species by exercise [34]. Misu et al. reported that selenoprotein P in the liver, which has antioxidative activities, was increased in T2D patients and contributed to the elimination of the beneficial effects of exercise on muscle [35]. Although the mechanism underlying the decreased oxidative stress in the soleus muscle of SDT fatty rats remains unknown, a certain degree of oxidative stress may be needed to maintain muscle mass and force mainly by type I oxidative fibers. Oxidative stress is not a common aggravating factor related to the progression of sarcopenia and DKD.

The results of the present study may be limited by several factors. First, we found no independent association between DKD and muscle atrophy in the SDT fatty rats. This point needs to be clarified in further studies aimed to determine whether muscle atrophy is prevented along with treatment of DKD. Second, while muscle strength decreases along with age and develops to sarcopenia in humans, muscle strength slightly increased during the experimental period and retention of muscle growth was observed in the SDT fatty rats from 16 to 24 weeks of age. Therefore, sarcopenia in humans was not completely reproduced in the present study; thus, further clinical studies of T2D patients are needed in order to reveal the clinical usefulness of urinary L-FABP as a marker of the progression of sarcopenia. Third, we did not determine the most suitable marker for sarcopenia because sample size was limited in the basic research. Future clinical research is needed to reveal the point. Finally, we did not investigate changes in urinary L-FABP levels in response to interventions that ameliorate renal hypoxia. Finally, we did not investigate changes in urinary L-FABP levels in response to interventions that prevent muscle atrophy. Because we recently demonstrated that habitual exercise led to a reduction in urinary L-FABP levels in healthy middle-aged and older adults [11], urinary L-FABP may respond to the relief of secondary sarcopenia due to diabetes and kidney disease.

## 5. Conclusion

In conclusion, urinary L-FABP reflects the degrees of muscle strength, muscle weight, and cross-sectional areas of muscle fibers. Although the detailed mechanisms of the interaction between sarcopenia and DKD was not revealed in this study, urinary L-FABP may be useful to monitor the progression of sarcopenia in addition to DKD in T2D patients. Nonetheless, future clinical studies of T2D patients are needed.

## Figures and Tables

**Table 1:** Time-related changes in body weight, blood glucose, systolic blood pressure, and food intake per weight.

Variable	Species	16weeks of age	20 weeks of age	24 weeks of age
Body weight (g)	SD	549.5 (12.8)	616.3 (20.8)	643.3^‡^ (19.4)
	SDT	546.7 (25.5)	574.9 (31.0)	580.0 (39.7)

Blood glucose (mg/dl)	SD	67.3 (6.1)	61.6 (3.6)	63.3 (3.3)
	SDT	199.6^∗∗^ (61.7)	184.0^∗∗^ (70.3)	215.6^∗∗^ (78.0)

Systolic blood pressure (mmHg)	SD	112.0 (5.1)	127.6 (5.8)	127.9 (3.7)
	SDT	120.6 (4.8)	131.0 (4.0)	140.6 (5.6)

Food intake per weight (g/kg)	SD	22.6 (4.7)	46.3^†^ (2.1)	47.1^†^ (1.4)
	SDT	74.4^∗∗^ (7.1)	67.0^∗^ (8.2)	47.1 (10.9)

Data are presented as the mean (standard error). ^∗^*p* < 0.05 and ^∗∗^*p* < 0.01 vs. the SD rats at the same week; ^†^*p* < 0.05 and ^‡^*p* < 0.01 vs. the same group at 16 weeks of age.

**Table 2:** Serum parameters at 24 weeks of age.

Variable	SD rat	SDT fatty rat
Serum creatinine (mg/dl)	0.45 (0.02)	0.55 (0.02)^∗∗^
Cystatin C (ng/ml)	3.68 (0.16)	4.01 (0.17)
Urea nitrogen (mg/dl)	18.79 (0.35)	29.42 (2.71)^∗∗^
Insulin (*μ*U/ml)	0.91 (0.29)	0.89 (0.21)
IGF-1 (pg/ml)	1199 (40.5)	826 (73.00)^∗∗^
Total cholesterol (mg/dl)	75.25 (3.92)	161.50 (6.94)^∗∗^
Triglyceride (mg/dl)	68.86 (4.09)	470.0 (119.00)^∗∗^

Data are presented as the mean (standard error). ^∗∗^*p* < 0.01 vs. the SD rats.

**Table 3:** Relationships between renal morphological change and muscle strength, and weights of the soleus and extensor digitorum longus (EDL) muscles in 24-week-old SD and SDT fatty rats.

Renal interstitial change	Muscle strength and weight	Correlation coefficient (*ρ*)
Macrophage infiltration	Muscle strength	-0.76^∗∗^
	Weight of soleus muscle	-0.67^∗^
	Weight of EDL muscle	-0.64^∗^
*α*-SMA expression	Muscle strength	-0.69^∗^
	Weight of soleus muscle	-0.67^∗^
	Weight of EDL muscle	-0.61^∗^
Collagen type I expression	Muscle strength	-0.57
	Weight of soleus muscle	-0.67^∗^
	Weight of EDL muscle	-0.62^∗^
Collagen type III expression	Muscle strength	-0.58
	Weight of soleus muscle	-0.83^∗^
	Weight of EDL muscle	-0.70^∗∗^
Glomerular sclerosis score	Muscle strength	-0.79^∗∗^
	Weight of soleus muscle	-0.49
	Weight of EDL muscle	-0.71^∗^

^∗^p < 0.05 and ^∗∗^p < 0.01.

## Data Availability

The data used to support the findings of this study are included within the article.
